# Can working memory account for EMDR efficacy in PTSD?

**DOI:** 10.1186/s40359-022-00951-0

**Published:** 2022-11-01

**Authors:** Dany Laure Wadji, C. Martin-Soelch, V. Camos

**Affiliations:** 1 I-Reach Lab, Unit of Clinical and Health Psychology, Department of Psychology, University of Fribourg, Fribourg, Switzerland; 2 W-MOVE (Working meMOry deVElopment) lab, Department of Psychology, University of Fribourg, Fribourg, Switzerland CH-1700; 3 Department of Psychology, University of Fribourg, Rue P.-A.-de Faucigny 2, CH-1700 Fribourg, Switzerland

**Keywords:** Post-traumatic stress disorder (PTSD), Eye Movement Desensitization and Reprocessing (EMDR), Working memory, Dual taxation, Eye movement, Bilateral stimulation

## Abstract

**Background:**

Although eye movement desensitization and reprocessing (EMDR) has been shown to be effective in the treatment of PTSD for years, it remains controversial due to the lack of understanding of its mechanisms of action. We examined whether the working memory (WM) hypothesis –the competition for limited WM resources induced by the dual task attenuates the vividness and emotionality of the traumatic memory – would provide an explanation for the beneficial effect induced by bilateral stimulation.

**Methods:**

We followed the Prisma guidelines and identified 11 articles categorized in two types of designs: studies involving participants with current PTSD symptoms and participants without PTSD diagnosis.

**Results:**

Regardless of the types of studies, the results showed a reduction of vividness and emotionality in the recall of traumatic stimuli under a dual-task condition compared to a control condition, such as recall alone. However, two studies used a follow-up test to show that this effect does not seem to last long.

**Conclusion:**

Our results provide evidence for the WM hypothesis and suggest that recalling a traumatic memory while performing a secondary task would shift the individual’s attention away from the retrieval process and result in a reduction in vividness and emotionality, also associated with the reduction of symptoms.

## Introduction

Post-traumatic stress disorder (PTSD) is a mental disorder that can occur in response to a traumatic or life-threatening event [1]. The prevalence rates of PTSD are between 1.9% and 8.8% [2]. PTSD results in significant social and economic burden and puts individuals at increased risk for physical and mental health problems [3]. Symptoms of PTSD include intrusive memories or recollections (e.g., flashbacks or nightmares), persistent avoidance of stimuli reminiscent of the traumatic event, negative alterations in cognition and mood, and hyperarousal or changes in physical and emotional reactions (e.g., restricted range of affect, feelings of detachment and disinterest) [1, 4, 5]. Patients with PTSD often describe difficulties with concentration, attention, and memory [6–8]. Many studies have documented poorer performance on tests of attention, working memory (WM), and other cognitive domains including mental manipulation and retroactive interference [6, 8, 9].

Clinical practice guidelines recommend several evidence-based treatments for PTSD, including the use of eye movement desensitization and reprocessing (EMDR) [10]. The efficacy of EMDR is now well established [11–14]. However, the mechanisms underlying its efficiency remain unclear.

Since its introduction, EMDR has been subject to debate [15]. In particular, the necessity of bilateral stimulation like eye movement (EM) during EMDR is controversial. Recent meta-analysis by [16] suggests that bilateral stimulation can be considered as a distraction inducing an attention-demanding task, which is (at least partially) necessary for an efficient treatment by EMDR. Other type of stimulations apart from EM that have been found to be beneficial include drawing a Figure [17], subtraction arithmetic [18], and playing the computer game Tetris [19].

A central component of EMDR treatment is the dual focus of attention, also called dual taxation [15]. Dual taxation takes place when an individual recalls a distressing memory while also performing a bilateral stimulation or secondary task, such as EM, mental arithmetic, or drawing complex Figure [20]. During the session, the therapist works on memory retrieval while getting the patient to engage in a secondary task [21, 22]. It can be suggested that the secondary task distracts attention away from the retrieval of memory. This reduction in attention would then impact the mechanisms of retrieval, disturbing it and resulting in an incomplete retrieval of memory traces. In the case of PTSD, memory traces of the traumatic event are characterized by the vividness of the emotion. The partial retrieval of these memory traces would blur their content, resulting in the reduction of vividness or the level of emotion linked to the memory traces. This partial retrieval thus leads to the reprocessing of the memory traces. Considering the functioning of the cognitive system, the present work specifically examined WM as one potential mechanism of EMDR action, with the hypothesis that this reprocessing takes place in WM. Other putative mechanisms of action of EMDR, such as mimicking of rapid eye movement (REM) states and the orientating response hypothesis[23] as well as the integration of the traumatic event into semantic networks[24] for instance will not be investigated in this article.

WM is the cognitive structure in charge of the short-term storage of information in view of its processing. Its dual function (storage and processing) has been extensively examined since the earlier study by [23]. Recently, a consensus emerged in the WM literature that this dual function depends on the allocation of attention (see 24 for review). Although the different WM models diverge on how exactly attention is shared between storage and processing, ample evidence was gathered to support the idea that any distraction of attention by a secondary task has a detrimental effect on storage (see 25 for review). More significant to the topic of the present study, the competition in attention allocation created by the dual task impairs memory retrieval; as such, the disturbing images would become less emotional and vivid [15, 17, 26–30]. This could be a potential mechanism of action of EMDR treatment for PTSD, and this hypothesis has been supported by several studies [17, 27, 31, 32]. However, there is still no systematic review examining this hypothesis – the WM hypothesis – as a mechanism supporting EMDR treatment.

Therefore, the purpose of this study is to summarize the state of research about the role of dual tasking during EMDR treatment for PTSD. We propose an interdisciplinary perspective integrating cognitive science and clinical psychology to improve our comprehension of the mechanisms underlying the effects of EMDR with a focus on working memory. Our specific objective is to examine whether the WM hypothesis – that competition for limited WM resources (i.e., attention) induced by the dual task attenuates the vividness and emotionality of the traumatic memory – would provide an explanation for the beneficial effect induced by eye movement or other bilateral stimulation during EMDR treatment for PTSD.

## Method

### Search strategy

**Fig. 1:**
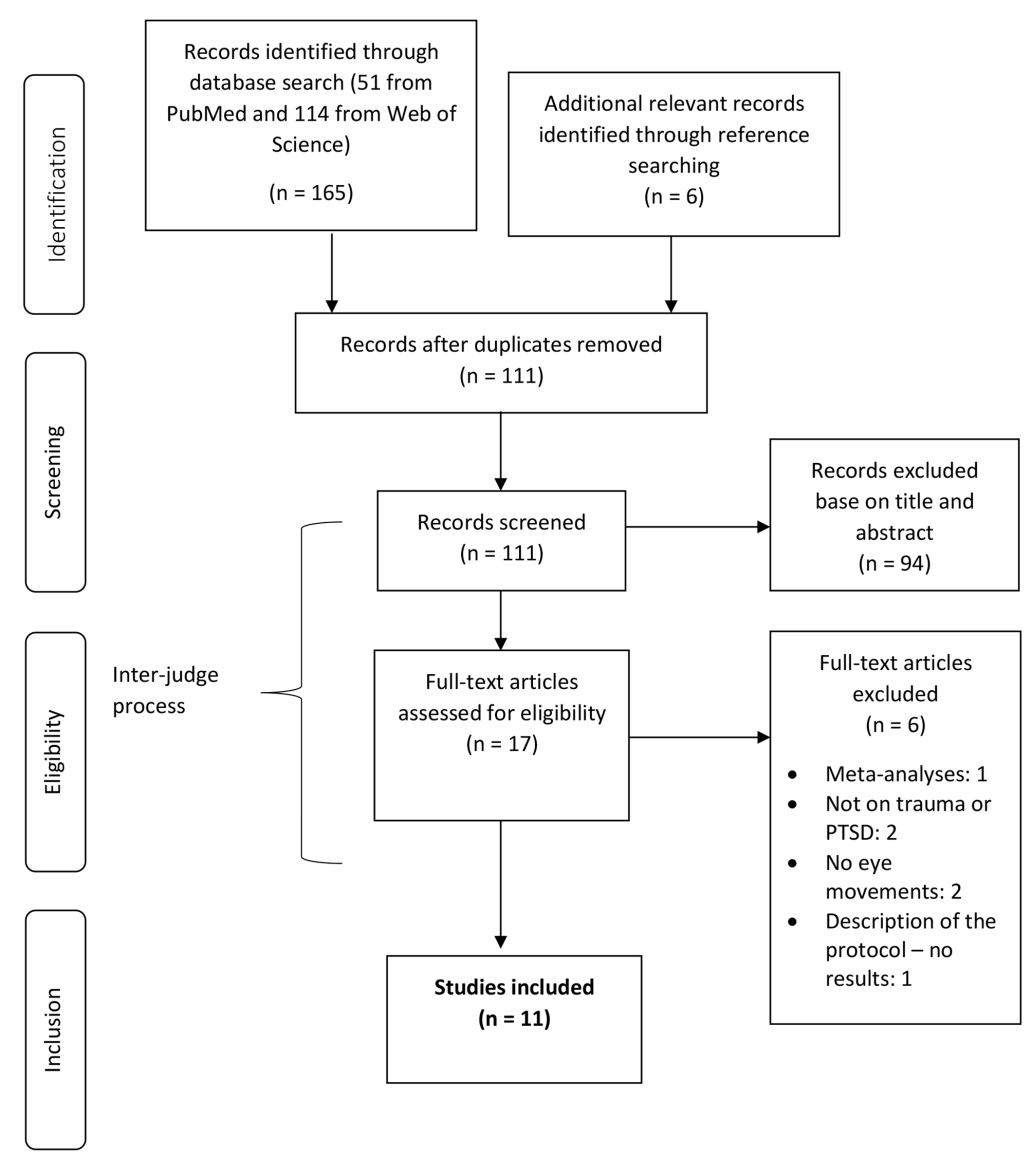
PRISMA flowchart of the identification and selection of studies PTSD: post-traumatic stress disorder

The searching and reporting of results followed the Preferred Reporting Items for Systematic Reviews and Meta-Analyses (Prisma) guidelines [33]. A systematic search was conducted in PubMed and Web of Science on the role of WM in EMDR from 1987 (the starting point of Shapiro’s work on EMDR) up to 21 March 2021. The search term was ‘EMDR and working memory’. We chose keywords with large meanings in order to reach the maximum number of results. The screening, with respect to PTSD, was done in a second step during the screening and selection of studies, during which all articles not related to PTSD were excluded. The automatic search was completed with a manual search from the references list of previous systematic reviews, meta-analysis, and the retrieved articles. Our search yielded 165 records: 114 in Web of Science, 51 in PubMed, and 6 from additional relevant records (see Fig. 1).

### Inclusion and exclusion criteria

The inclusion criteria were as follows: (i) studies must report empirical evidence published in peer-reviewed journals; (ii) they must be written in English; (iii) they must focus on the case of PTSD or PTSD-induced conditioning; (iv) they must examine the mechanism of action of EMDR; (v) they must focus on dual taxation of the working memory; and, finally, (vi) they must focus on eye movement and other forms of bilateral stimulation (e.g., tactile, sound).

We excluded: (i) book reviews, presentations, theses, literature reviews, meta-analyses, and magazines; (ii) papers published in a language other than English; (iii) papers that were not on trauma, PTSD, or PTSD-induced conditioning.

### Screening and eligibility

After removing the duplicates, two reviewers (D. W. and V. C.) independently screened the studies at two different stages: title/abstract and full text. This was to ensure that no potential articles were left out and that the articles included in the review fulfilled the inclusion criteria. The percentage of consensus between the two reviewers was about 80%. Disagreements were resolved by discussion and consensus based on inclusion and exclusion criteria.

### Data processing, extraction, and synthesis

One author (D. W.) extracted the main information from the included studies, and this information was independently reviewed and cross-checked for accuracy and completion by the other author (V. C.). Finally, we also reported the statistical results in terms of test value or effect size, as well as the results of the statistical tests performed.

## Results

### Descriptive summary

We identified a total of 171 studies, of which 111 remained after filtering out duplicates. Titles and abstracts were then screened; of these, 94 were excluded for the following reasons: was not empirical (e.g., reviews, n = 17), was a thesis (n = 2), was not on post-traumatic stress disorder (n = 45), was not on EMDR (n = 5), did not examine WM (n = 23), was a case study (n = 1), or was written in a language other than English (n = 1). We read the full text of 17 studies that appeared to meet the inclusion criteria to assess them for eligibility. Six papers were removed at this stage due to the following reasons: was not on trauma or PTSD (n = 2), was not on eye movements (n = 2), was a description of the protocol without results (n = 1), and was a meta-analysis (n = 1). A total of 11 original reports published between 2011 and 2021 were included in the systematic review. The full details of these studies are provided in Table 1.

**Table 1:** Summary of articles included in the systematic review (11 studies)

Authors	Objectives	Participants			PTSD diagnostic tools	Study design			Results
		**Type and number**	**Experimental group**	**Control group**		**Type of study**	**Conditions**	**Measures**	
(Engelhard, van den Hout, Dek, et al., 2011)	Investigate whether double task could provide better desensitization of the traumatic memory	Participants with recurrent intrusive/ disturbing visual images (N = 37)	1 group (N = 37)	No control group	Yale-Brown Obsessive Compulsive Scale and SCL-90	Experimental study using computer and behavioural task/measure of an emotionally disturbing auditory memory	Two conditions: recall intrusive images with EM or recall without making eye movements	VAS score on vividness and emotionality	Vividness of intrusive images was lower after recall with eye movement, relative to recall only (t(36) = 2.37, p < .05, d = 0.37), and there was a similar trend for emotionality (t(36) = 2.01, p < .05, d = 0.32).
(Thomaes et al., 2016)	Examine visual and emotional processing brain regions as well as the activity of the DLPFC during the recall of the traumatic memory recall, with EM relative to recall-only	Participants with current PTSD symptoms (N = 8)	1 group (N = 8)	No control group	Structured clinical interview and PSS-SR scale	Experimental study using functional magnetic resonance imaging	Two conditions: recall of the traumatic memories with EM and recall without EM	Neural activation in brain regions in response to memory recall during script-driven imagery	EM during recall, compared to recall only, was associated with reducing activity – i.e., less activation of right amygdala and rostral ACC and connectivity in emotional processing in brain regions (T = 3.499, p-uncorrected < 0.005)
(Sack et al., 2016)	Examine if there is better desensitization with exposure alone, with a double task, or with a visual fixation task	Participants with current PTSD (N = 139)	EM group (N = 47) and EF group (N = 47)	Control group (EC) (N = 45)	CAPS interview based on the DSM-IV	Randomized clinical trial	Three conditions: eyes moving on the therapist’s moving hand (EM), eyes fixating on the therapist’s non-moving hand (EF), and exposure without explicit visual focus of attention as the control condition (EC)	CAPS scores on PTSD symptoms and remission of PTSD diagnosis after EMDR session	Larger symptom decrease in EM and EF than in EC (CAPS: EM = 35.8, EF = 40.5, EC = 31.0) and significantly larger effect sizes (EM: d = 2.06, 95% CI: 1.55–2.57, EF: d = 2.58, 95% CI: 2.01–3.11, EC: d = 1.44, 95% CI: 0.97–1.91); no difference between EM and EF
(Matthijssen et al., 2017)	Investigate whether auditory memories can be targeted with EMDR in PTSD patients	Participants with current PTSD symptoms (N = 30)	1 group (N = 30)	No control group	Clinical psychologist/psychiatrist screening using DSM IV-TR criteria	Experimental study with behavioural task/measure of an emotionally disturbing memory	Three conditions: to make EM (VT), to count down (AT), or to stare at a non-moving dot (CC)	SUD score on the emotionality of a disturbing image and reaction time	Emotionality of auditory memory was reduced in the three conditions; however, no difference was found between AT, VT, or the CC [Auditory Memory: [F(2,58) = 2.02, p = .14]; Visual Memory F(2,60) = 0.25, p = .78]
(Matthijssen et al., 2019)	Examine the extent to which emotionality of auditory hallucination memories could be reduced by dual tasking	Participants with current PTSD symptoms (N = 36)	1 group (N = 36)	No control group	PSYRATS-AH questionnaire, BAVQ-R questionnaire, SUD score	Experimental study with behavioural task/measure of an emotionally disturbing auditory memory	Three conditions: visual taxation though EM (VT), auditory taxation by counting out loud (AT), and control condition without any additional task (CC)	SUD score on the emotionality of a disturbing image	The active conditions – i.e., making eye movements or counting out loud – showed stronger effects in reducing emotionality of auditory hallucinations compare to the control condition [BF1 = 5.8, model 1: AT (pre-post) = VT (pre-post) > CC (pre-post)]
(Matthijssen & van Hout, 2016)	Examine the effects of eye movements on positive verbal imagery after an EMDR session	Healthy participants (N = 30)	1 group (N = 30)	No control group	\	Experimental study using selected individual negative memory	Two conditions: EM and eyes stationary (ES)	VAS scale on the belief in personality trait and perception checklist scores	No significant differences between the eye movement and the eyes stationary conditions; eye movements did not diminish or enhance the belief of the positive relevant personality trait (F(1, 35) = 0.071, p = .792)
(Voogd & Phelps, 2020)	Examine whether the impact of a working memory task on extinction learning is greater when cognitive load is increased	Healthy participants exposed to PTSD-induced trauma (n = 75) across three groups	Low-load group (N = 24) and high-load group (N = 27)	Control group (N = 24)	\	Experimental study with induced PTSD using images of snakes associated with peripheral stimulation	Three conditions: low-load condition group, high-load condition group, and without dual task as the control condition	Accuracy scores on a random sequence, SCR response, and reaction times	The conditioned response was stronger in the control group compared to the low-load group [F(1, 46) = 4.24, p = .045, ηp 2 = 0.08] and the high-load group [F(1, 49) = 12.07, p = .001, ηp 2 = 0.20]; also, a stronger cognitive load had a bigger impact on the reduction in the conditioned response compared to the control condition [t(72) = − 3.619, p < .001]
(Leer & Engelhard, 2020)	Examine the effect of induced EM on memory accuracy on a visual discrimination task	Healthy participants (n = 68)	Group for discrimination test day 2 (*N* = 34) and group for discrimination test day 1 and day 2 (*N* = 34)	No control group	\	Experimental study with induced trauma using two sets of images and a 2-ms electrocutaneous stimulus	Two conditions: recall with EM condition vs. recall without eye movements condition	Shock occurrence prediction, shock expectancy rating score, number of false positive responses, and reaction time of responses	False positive rates in a discrimination task increased in recall with eye movement condition the day after the conditioning phase, compared to the control condition (F(1, 66) = 14.58, p < .001, ηp2 = 0.181)
(van Schie et al., 2019)	Investigated the effects of dual tasks on intrusive memories following analogue trauma	Healthy participants (n = 76)	1 group (N = 76)	No control group	\	Experimental study with induced trauma using word-image association pairs	Three conditions: recall + EM condition, recall + counting condition, and no task (control condition); two different dual tasks to quantify WM (RIR + EM, RIR + counting, or RIR only	VAS score on vividness and emotionality, response latency score, and choice confidence	Cognitive loads of RIR + EM and RIR + counting were higher than RIR only (BFs10 > 2.81 × 10^17^); hotspot vividness and unpleasantness ratings were not affected by the intervention; WM taxation was not related to decreases in vividness (r = − .19, BF01 = 12.60) or unpleasantness (r = − .04, BF01 = 6.90)
(van Veen et al., 2020)	Examine if recall + EM results in larger immediate and 24-hour reductions in memory vividness, negative valence, and distress than recall alone	Healthy participants (n = 45)	Recall + EM group (N = 50) and recall only group (N = 50)	No control group	\	Experimental study with induced trauma using negative autobiographical memory	Two conditions: recall with EM vs. recall without EM	VAS score on vividness, negative valence, and distress ratings; SUIS score on tendency and ease of forming visual images in daily life; ACS score; automated reading span	After four sessions, memory was deflated in recall with eye movements –vividness − 0.90 (− 3.10–1.23); negative valence 0.64 (− 0.76–2.14); distress 0.82 (− 1.17–2.86); memory was inflated in recall alone –vividness − 2.84*(− 4.95 to − 0.71), negative valence − 2.13*(− 3.60 to − 0.70), distress − 3.59*(− 5.57 to − 1.58) After 32 sessions, there was a reduction in both recall conditions for all outcome measures
(Rackham & Lau-Zhu, 2021)	Investigate whether mental imagery of the 9/11 terrorist attacks following media exposure is dampened by taxing working memory	Healthy participants (n = 45)	1 group (N = 45)	No control group	\	Experimental study with PTSD induced using a personally relevant mental image of the 9/11 terrorist attacks	Three conditions: recall + EM, recall + Tetris game, and recall only	11-point Likert scale on imagery vividness and emotionality	Compared to recall only, dual-task effects (i.e., recall + Tetris and recall + EM) reduced ratings of vividness and emotionality – recall + Tetris, F(1, 28) = 27.90, p < .001, h2 p = .50, 90% CI = [0.26, 0.64]; recall + EM, F(1, 28) = 37.44, p < .001, h2 p = .57, 90% CI = [0.34, 0.69]; this effect vanished in a follow-up (24 h later) test –recall + Tetris, t(14) = 4.75, p < .001, d = 1.22, and recall + EM, t(14) = 5.82, p < .001, d = 1.51)

### Characteristics of included studies

All 11 studies used a design comparing different conditions (i.e., with vs. without dual task) and/or groups (i.e., control and experimental group). Among the 11 studies, we identified two different types of designs: studies involving participants with current PTSD symptoms (N = 5: [31, 34–37] and studies testing healthy participants (N = 6) – that is, without a PTSD diagnosis or current psychiatric disorder – but who were exposed to potential traumatic stimuli by means of trauma films [38] or pictures or images [32, 39], or who were called upon to remember a traumatic event [40–42]. The results are presented and organized according these two categories. We provide an addition paragraph on the long-term effect of the dual task intervention.

### Studies with participants having current PTSD symptoms

We found a total of five studies with participants having current PTSD symptoms, recruited mostly from specialized mental health care centres [31, 34, 36, 37]. One study recruited university students suffering from intrusive images determined by a therapist to be suitable for EMDR processing [35]. Two studies included participants having symptoms documented in patients with post-traumatic stress disorder, like intrusive images about potential future catastrophes – i.e., flashforwards [35] and auditory hallucinations [37], while three other studies included PTSD patients diagnosed by a trained clinician (e.g., clinical psychologist/psychiatrist) and based on Diagnostic and Statistical Manual of Mental Disorders (DSM) IV-TR criteria [31, 34, 36].

PTSD symptoms were assessed with instruments like the Yale-Brown Obsessive Compulsive Scale, with items 2 and 3 pertaining to the occurrence of intrusions and the degree of annoyance with them [35]; the Clinician-Administered PTSD Scale interview, based on the DSM-IV [31, 34, 36]; and the Psychotic Symptoms Rating Scale–Auditory Hallucinations questionnaires [37].

After the experiment, most studies indicated a better desensitization of the traumatic memories or images in terms of reduced vividness and emotionality under the dual-task conditions compared to the control condition. For example, 35 showed that dual task (i.e., recall with EM) was associated with reduction in the vividness and emotionality of intrusive images, compared to recall without EM. In the same vein, 31 reported reduced activity and reduced connectivity in emotional processing in brain regions using functional MRI measures. They found that right amygdala and rostral anterior cingulate cortex (ACC) activity was significantly lower after recall with EM than after recall only. Similarly, functional connectivity from amygdala to rostral ACC was decreased after recall with EM compared to recall only. 34 performed a randomized clinical trial and observed better desensitization for the dual-task conditions (i.e., exposure with eyes moving while fixating on therapist’s moving or non-moving hand), with a larger decrease of symptoms and a high remission rate of PTSD diagnosis compared to exposure alone. Finally, 37 showed that making eye movements or counting out loud resulted in a stronger reduction in the emotionality of auditory hallucinations compared to the control condition.

However, the study by36 showed that the emotionality of auditory memories was reduced in the three conditions: making EM (visual taxation), counting down (auditory taxation) or staring at a non-moving dot (control condition), with a stronger reduction under the auditory and visual taxation conditions than under the control condition. Nevertheless, they found no significant difference between the three conditions, indicating no modality-specific effect and no support for the efficacy of dual taxation.

It is important to note that most studies were based on self-reported behavioural tasks as an outcome measure – in particular, the visual analog scale (VAS) score on vividness and emotionality of disturbing memories or images [34–37]. Such a measure can be prone to bias. Only one study, by 31, used functional MRI measures. Furthermore, the sample size varied considerably, from 8 participants to 139 participants, and most studies did not include a control group.

Nevertheless, the overall consistency in findings is notable, supporting the beneficial effect of dual taxation. The results point to a reduction in the vividness of intrusive images and in their emotionality under dual-task conditions compared to the control condition. In addition, two studies [34, 43] also reported evidence for a larger decrease in symptoms and a high remission rate of PTSD diagnosis under the dual-task conditions.

### Studies with healthy participants without PTSD diagnosis but who were exposed to potential traumatic stimuli

Out of the 11 included studies, six studies examined healthy participants without PTSD symptoms but who were exposed to potential traumatic stimuli. Four studies reported a significant difference between the experimental conditions, supporting the hypothesis that there is a reduction in vividness and emotionality under dual-task conditions compared to the control condition. For instance, 32 showed how low- and high-cognitive load tasks reduced responses to threat and enhanced extinction learning compared to the control condition. Moreover, higher cognitive load had a bigger impact on the reduction in the conditioned response compared to the two other conditions (low load and control). Finally, the participants with the lower accuracy rate in the high-load task had the stronger reduction in conditioned response. 39 investigated the effect of induced EM on memory accuracy on a visual discrimination task with two conditions of recall (with vs. without EM) in a sample of 68 undergraduates. Their results showed that false positive rates increased in recall with EM the day after the conditioning phase. 40 examined whether recall with EM would result in larger reductions in memory vividness, negative valence, and distress than recall alone in a sample of 45 participants. The participants recalled and rated negative autobiographical memories in eight successive blocks of four sessions (lasting 24 s each). Memory was deflated after the first block of four sessions in a recall test with EM, but it was inflated under the condition with recall alone. Lastly, 42 investigated whether mental imagery of the 9/11 terrorist attacks following media exposure was dampened by taxing WM in 45 young healthy adults. They compared three conditions of recall – with EM, with a concurrent task (playing Tetris), or with no additional task (recall only). Compared to the recall-only control condition, dual-task conditions reduced ratings of vividness and emotionality.

However, two studies reported that dual-task conditions did not result in greater decreases in hotspot vividness/unpleasantness compared to a control condition. For example, 41 tested a non-clinical sample of 30 undergraduates and examined the effects of EM on positive verbal imagery after an EMDR session using two conditions of recall: with 15–20 s of horizontal EM or with 15 s of eyes stationary control condition. Their results showed no significant differences between the two conditions. 38 investigated 76 healthy students on the effects of dual tasks on intrusive memories after watching a trauma film using three conditions: recall with EM, recall with a concurrent task, or recall without any additional task (control condition). Even after prolonging the duration of the intervention (from 6 × 24s in the first experiment to 16 × 24s in the second experiment) and adding additional measures for intrusion characteristics, they found no effect for the dual task, because recall with EM or with a concurrent task did not lead to greater drops in vividness and unpleasantness compared to the absence of a task in the control condition. As such, both studies showed possible methodological limitations related to the use of subjective measures, as well as to issues related to whether the intervention should be short or long.

Overall, except for two studies that failed to report a significant effect of dual task, the results obtained from healthy participants exposed to potential traumatic stimuli support a reduction in vividness and emotionality during a short period.

### Long-term effect of the dual task intervention

Two studies with healthy participants without a PTSD diagnosis but who were exposed to potential traumatic stimuli also included information on follow-up tests. 40 showed that after the 4 × 24s intervention, recall under EM resulted in memory deflation. Meanwhile, recall only (i.e., without EM) caused memory inflation. However, after the full intervention (32 × 24s), both conditions resulted in immediate and 24-hour reductions on all outcome measures. 42 also showed that, compared to the recall-only control condition, dual-task conditions reduced ratings of vividness and emotionality; however, this effect vanished in a follow-up test that took place 24 h later.

In conclusion, there is a reduction in the ratings of vividness and emotionality after a short intervention, but this effect of dual task disappears with time, as evidenced by the follow-up tests.

## Discussion

**Fig. 2:**
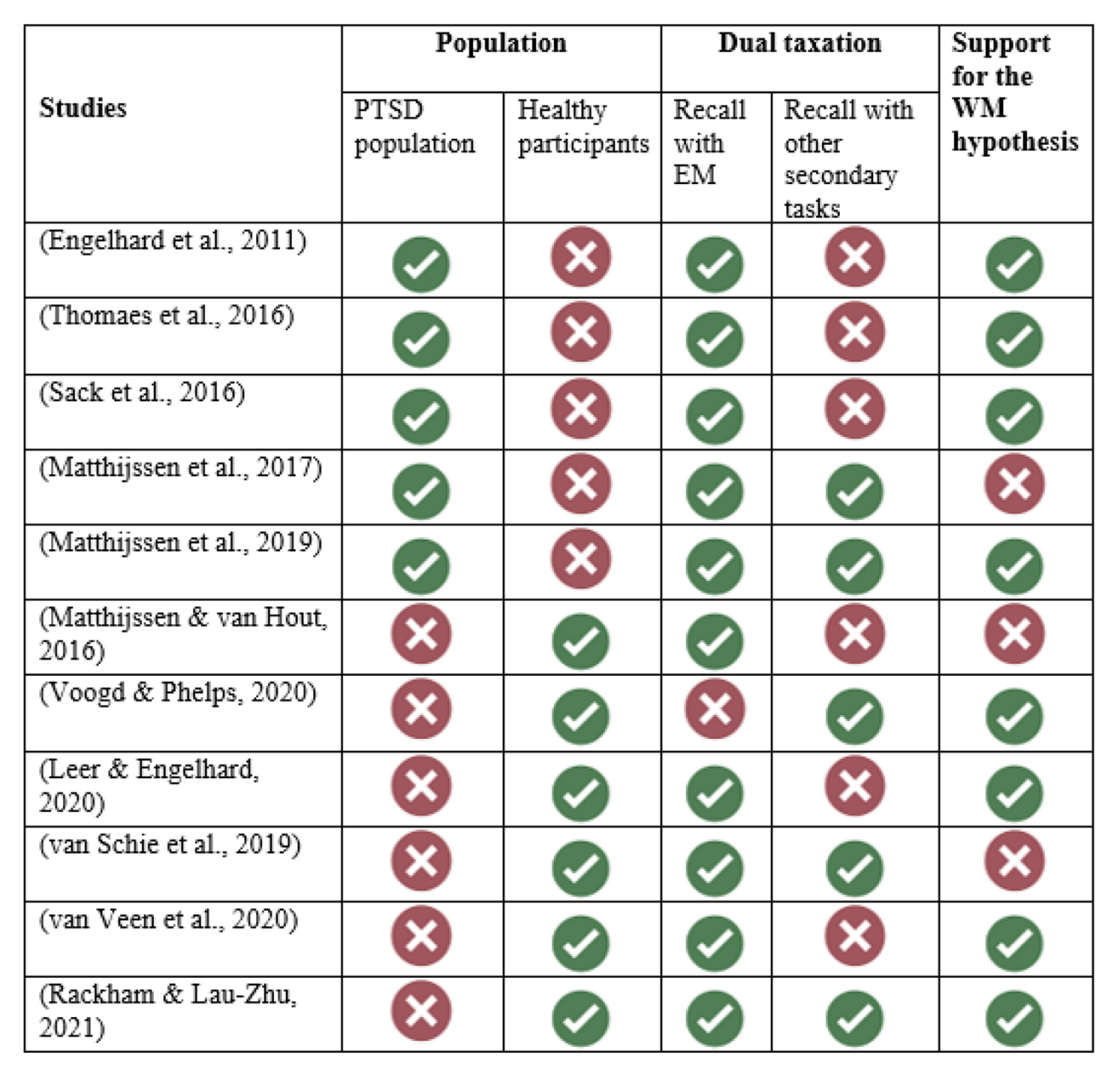
Graphically summary of the results Note: Post-traumatic stress disorder (PTSD), eye movements (EM), working memory (WM)

The purpose of this systematic review was to examine whether the WM taxation model provides an explanation for the beneficial effect induced by EM in EMDR treatment. We identified 11 studies that met our inclusion criteria and categorized them into two sets: studies with participants having current PTSD symptoms and studies with healthy participants without a PTSD diagnosis who were exposed to potential traumatic stimuli. The most remarkable finding of this systematic review is that, overall, regardless of the type of studies, the results showed a reduction of vividness and emotionality in the recall of traumatic stimuli under a dual-task condition compared to a control condition such as recall alone (see Fig. 2). Our results showed (a) that a secondary eye movement task during recall weakens emotional memory retrieval, (b) that secondary tasks that do not involve eye movements (e.g., counting, playing Tetris) weaken retrieval to a similar degree, and (c) that competing demands on working memory are the cause of these effects. However, two studies using a follow-up test showed that this effect does not seem to last long, as it disappeared over time.

In line with our hypothesis, we found a reduction in vividness and emotionality in the recall of traumatic stimuli under dual-task conditions compared to a control condition. Interestingly, this result is fairly similar for participants having current PTSD symptoms and for healthy participants who were experimentally exposed to traumatic stimuli. Notably, the more complex the secondary task during EMDR is, the greater the reduction in vividness and distress associated with negative memories. Most of the studies showed that the variation in the difficulty level of the secondary task plays a role in the efficient desensitizing of the traumatic memory. For example, doing EM, counting, and playing Tetris during the recall of a disturbing memory or image showed stronger effects than the control condition – i.e., without any secondary tasks. Our results provide support for the WM hypothesis as an account for the functioning of EMDR. The recall of memory or stimuli competes with the secondary task (e.g., EM, counting, or playing Tetris) for limited WM capacity. The resource taxation by the secondary task would deflate some aspects of the memory. As a consequence, the memory would be blurred during the dual task, and the disturbing images would become less emotional and vivid [15, 26, 40].

With regard to trauma symptoms, we also found that, in studies with participants having current PTSD symptoms, there was a larger decrease in symptoms under the dual-task conditions compared to the control condition. Although no information was reported on whether the treatment outcome was stable over time, a randomized clinical trial study by 34 showed that remission of PTSD diagnosis after EMDR session and larger treatment effects were observed with dual taxation. This suggests that this diminution of symptoms could be related to the decrease in the vividness and emotionality of the traumatic stimuli.

Our results also suggest that the effects of dual taxation disappear with time, as shown by two studies that provided follow-up tests. However, it is important to take into account that these studies were conducted on healthy participants and not on participants with PTSD symptoms. More studies with follow-up tests are definitely warranted.

Most of the studies used self-reported evaluation scales to rate the vividness and emotional intensity of the disturbing memory, which may be prone to bias [44]. The most common behavioural outcomes measures used was the VAS score on vividness and emotionality, followed by the SUD score. However, only one study provides insight into the neurobiological mechanisms underlying the effects of dual taxation. 31 showed that EM during recall was associated with reduced activity in certain brain regions, such as less activation of the right amygdala and rostral anterior cingulate cortex (ACC), which serve as a central hub for cognitive and emotional networks [45, 46]. This study suggests a neurobiological pathway – i.e., the reduced activity of the amygdala and ACC – which may help to understand how dual taxation during EMDR leads to less vivid and less emotional traumatic stimuli. This would fit with previous studies showing a relationship between EM and attention regulation [47, 48]. Nevertheless, because of the small sample size in the EMDR studies, replication in a larger and independent sample is warranted.

Although these studies provide valuable information on the taxation of WM involved in EMDR, they also have a number of limitations. First, there are very few studies with participants having current PTSD symptoms (n = 5). In addition, measures used to examine PTSD were quite different and included only partially symptoms related to PTSD and / or were not always aimed at diagnosing PTSD. This statistically limits the power of the conclusion. Second, most studies did not control for or determine reaction time, described as an objective index of the extent to which different dual attention tasks tax WM [49]. Knowing the reaction time could help understand the processing of information and appreciate the duration of mental processes of various conditions, as suggested by [50]. The fact that most studies did not control for reaction time does not allow for conclusions about the causal nature of cognitive variables. Third, the use of truly experimental design is limited in studies of PTSD patients. For ethical reasons, it is very difficult to experimentally manipulate how traumatic events are processed. Fourth, in most cases, the assessment of vividness and emotion was conducted using self-reports of behavioural measures, which may be prone to bias and self-representation strategies. Fifth, only two studies provided a follow-up test, thus making it difficult to draw conclusions on the efficiency of dual taxation over time.

## Clinical implications

From a clinical perspective, the conclusion of this systematic review may help advance the understanding of mechanisms involved in EMDR. Our results provide evidence for the WM hypothesis that EMDR efficacy is related to the distraction of attention during the retrieval of memory traces in WM. Recalling a traumatic memory while performing a secondary task would shift the individual’s attention away from the retrieval process and result in a reduction in vividness and emotionality, which is also associated with the reduction of symptoms. Thus, any task requiring attentional resources, whether visual or auditory, could allow for the desensitization of a traumatic memory, as well as its reprocessing in memory with a less intense and less vivid emotion. However, one must be very careful about the level of difficulty of the distractive task used. Caution should also be taken regarding the duration of these changes, since two studies showed that these effects disappeared over time.

## Conclusion

Although EMDR has been shown to be effective in the treatment of PTSD for years, it remains controversial due to the lack of understanding of its mechanisms of action. We propose an interdisciplinary perspective integrating cognitive science and clinical psychology to improve our comprehension of the mechanisms underlying the effects of EMDR with a focus on working memory. We examined whether the WM hypothesis provides an explanation for the beneficial effect induced by bilateral stimulation during EMDR therapy for PTSD. A notable finding of this review is that most studies indicated a reduction in the evaluation of the vividness and emotionality of the traumatic stimuli after EMDR in the healthy sample as well as in the PTSD sample. Overall, the studies examined provide support for the WM hypothesis. Our research paves the way for future studies to investigate this mechanism in a large sample and in a clinical setting using follow-up tests.

## Data Availability

The datasets used and analysed during the current study are available from the corresponding author on reasonable request.

## List of abbreviations

ACC: Anterior cingulate cortex
DSM: Diagnostic and Statistical Manual of Mental Disorders
EMDR: Eye movement desensitization and reprocessing
EM: Eye movement
FMRI: Functional magnetic resonance imaging
PTSD: Post-Traumatic stress Disorder
VAS: Visual analog scale
WM: Working memory

## References

[1] American Psychiatric Association (2013). Diagnostic and Statistical Manual of Mental Disorders, Fifth Edition (DSM-5®).

[2] Bisson JI, Cosgrove S, Lewis C, Robert NP (2015). Post-traumatic stress disorder. BMJ.

[3] Bomyea J, Johnson A, Lang AJ (2017). Information Processing in PTSD: Evidence for Biased Attentional, Interpretation, and Memory Processes. Psychopathol Rev.

[4] Shalev A, Liberzon I, Marmar C (2017). Post-Traumatic Stress Disorder. N Engl J Med.

[5] Shipherd JC, Salters-Pedneault K, Attention (2008). Memory, Intrusive Thoughts, and Acceptance in PTSD: An Update on the Empirical Literature for Clinicians. Cogn Behav Pract.

[6] Hayes JP, Vanelzakker MB, Shin LM (2012). Emotion and cognition interactions in PTSD: a review of neurocognitive and neuroimaging studies. Front Integr Neurosci.

[7] Neylan TC, Lenoci M, Rothlind J, Metzler TJ, Schuff N, Du A-T (2004). Attention, learning, and memory in posttraumatic stress disorder. J Trauma Stress.

[8] Vasterling JJ, Brailey K, Constans JI, Sutker PB (1998). Attention and memory dysfunction in posttraumatic stress disorder. Neuropsychology.

[9] Durand F, Isaac C, Januel D (2019). Emotional Memory in Post-traumatic Stress Disorder: A Systematic PRISMA Review of Controlled Studies. Front Psychol.

[10] World Health Organization. Guidelines for the management of conditions specifically related to stress. Geneva: WHO; 2013. Available from: URL: https://apps.who.int/iris/bitstream/handle/10665/85119/9789241505406_eng.pdf. 24049868

[11] Novo Navarro P, Landin-Romero R, Guardiola-Wanden-Berghe R, Moreno-Alcázar A, Valiente-Gómez A, Lupo W, et al. 25 años de Eye Movement Desensitization and Reprocessing: protocolo de aplicación, hipótesis de funcionamiento y revisión sistemática de su eficacia en el trastorno por estrés postraumático. Rev Psiquiatr Salud Ment 2018; 11(2):101–14. 10.1016/j.rpsm.2015.12.002 26877093

[12] Wilson G, Farrell D, Barron I, Hutchins J, Whybrow D, Kiernan MD (2018). The Use of Eye-Movement Desensitization Reprocessing (EMDR) Therapy in Treating Post-traumatic Stress Disorder-A Systematic Narrative Review. Front Psychol.

[13] Chen L, Zhang G, Hu M, Liang X (2015). Eye movement desensitization and reprocessing versus cognitive-behavioral therapy for adult posttraumatic stress disorder: systematic review and meta-analysis. J Nerv Ment Dis.

[14] Schnyder U, Ehlers A, Elbert T, Foa EB, Gersons BPR, Resick PA (2015). Psychotherapies for PTSD: what do they have in common?. Eur J Psychotraumatol.

[15] Landin-Romero R, Moreno-Alcazar A, Pagani M, Amann BL (2018). How Does Eye Movement Desensitization and Reprocessing Therapy Work? A Systematic Review on Suggested Mechanisms of Action. Front Psychol.

[16] Lee CW, Cuijpers P (2013). A meta-analysis of the contribution of eye movements in processing emotional memories. J Behav Ther Exp Psychiatry.

[17] Gunter RW, Bodner GE (2008). How eye movements affect unpleasant memories: support for a working-memory account. Behav Res Ther.

[18] Engelhard IM, van den Hout MA, Smeets MAM (2011). Taxing working memory reduces vividness and emotional intensity of images about the Queen’s Day tragedy. J Behav Ther Exp Psychiatry.

[19] Engelhard IM, van Uijen SL, van den Hout MA. The impact of taxing working memory on negative and positive memories. Eur J Psychotraumatol 2010; 1. 10.3402/ejpt.v1i0.5623 PMC3402003 22893797

[20] van Schie K, Engelhard IM, van den Hout MA (2015). Taxing Working Memory during Retrieval of Emotional Memories Does Not Reduce Memory Accessibility When Cued with Reminders. Front Psychiatry.

[21] Shapiro F, Laliotis D (2011). EMDR and the Adaptive Information Processing Model: Integrative Treatment and Case Conceptualization. Clin Soc Work J.

[22] Shapiro F (2001). Eye movement desensitization and reprocessing: Basic principles, protocols, and procedures. Eye movement desensitization and reprocessing: Basic principles, protocols, and procedures.

[23] Baddeley AD, Hitch G. Working Memory. In: Elsevier; 1974. pp. 47–89. (Psychology of Learning and Motivation).

[24] Logie RH, Camos V, Cowan N. The State of the Science of Working Memory. In: Logie RH, Camos V, Cowan N, editors. Working Memory. Oxford University Press; 2020. pp. 1–9.

[25] Barrouillet P, Camos V (2015). Working memory: Loss and reconstruction.

[26] van den Hout MA, Engelhard IM (2012). How does EMDR work?. J Experimental Psychopathol.

[27] Andrade J, Kavanagh D, Baddeley A (1997). Eye-movements and visual imagery: a working memory approach to the treatment of post-traumatic stress disorder. Br J Clin Psychol.

[28] Pagani M, Amann BL, Landin-Romero R, Carletto S. Eye Movement Desensitization and Reprocessing and Slow Wave Sleep: A Putative Mechanism of Action. Front Psychol 2017; 8:1935. 10.3389/fpsyg.2017.01935 PMC5681964 29163309

[29] Barrouillet P, Bernardin S, Camos V (2004). Time constraints and resource sharing in adults’ working memory spans. J Exp Psychol Gen.

[30] Camos V, Barrouillet P (2014). Le développement de la mémoire de travail: perspectives dans le cadre du modèle de partage temporel des ressources. Psychologie Française.

[31] Thomaes K, Engelhard IM, Sijbrandij M, Cath DC, van den Heuvel OA (2016). Degrading traumatic memories with eye movements: a pilot functional MRI study in PTSD. Eur J Psychotraumatol.

[32] de Voogd LD, Phelps EA (2020). A cognitively demanding working-memory intervention enhances extinction Research article. Sci Rep.

[33] Moher D, Liberati A, Tetzlaff J, Altman DG (2009). Preferred reporting items for systematic reviews and meta-analyses: the PRISMA statement. PLoS Med.

[34] Sack M, Zehl S, Otti A, Lahmann C, Henningsen P, Kruse J (2016). A Comparison of Dual Attention, Eye Movements, and Exposure Only during Eye Movement Desensitization and Reprocessing for Posttraumatic Stress Disorder: Results from a Randomized Clinical Trial. Psychother Psychosom.

[35] Engelhard IM, van den Hout MA, Dek ECP, Giele CL, van der Wielen J-W, Reijnen MJ (2011). Reducing vividness and emotional intensity of recurrent “flashforwards” by taxing working memory: an analogue study. J Anxiety Disord.

[36] Matthijssen SJMA, Verhoeven LCM, van den Hout MA, Heitland I. Auditory and Visual Memories in PTSD Patients Targeted with Eye Movements and Counting: The Effect of Modality-Specific Loading of Working Memory. Front Psychol 2017; 8:1937. 10.3389/fpsyg.2017.01937 PMC5675874 29163311

[37] Matthijssen SJMA, Heitland I, Verhoeven LCM, van den Hout MA (2019). Reducing the Emotionality of Auditory Hallucination Memories in Patients Suffering From Auditory Hallucinations. Front Psychiatry.

[38] van Schie K, van Veen SC, Hagenaars MA (2019). The effects of dual-tasks on intrusive memories following analogue trauma. Behav Res Ther.

[39] Leer A, Engelhard IM (2020). Side effects of induced lateral eye movements during aversive ideation. J Behav Ther Exp Psychiatry.

[40] van Veen SC, van Schie K, van de Schoot R, van den Hout MA, Engelhard IM (2020). Making eye movements during imaginal exposure leads to short-lived memory effects compared to imaginal exposure alone. J Behav Ther Exp Psychiatry.

[41] Matthijssen SJMA, van Hout M den. Fifteen to Twenty Seconds of Eye Movements Have No Effect on Believability of Positive Personal Verbal Statements: Results From a Working Memory Study. J EMDR Prac Res 2016; 10(2):82–90.

[42] Rackham LA, Lau-Zhu A (2021). Taxing working memory to modulate mental imagery of the 9/11 terrorist attacks following media exposure during childhood: a pilot study in young adult UK residents. Anxiety Stress Coping.

[43] Matthijssen SJMA, van Schie K, van den Hout MA (2019). The Effect of modality specific interference on working memory in recalling aversive auditory and visual memories. Cogn Emot.

[44] Lazaridou A, Elbaridi N, Edwards RR, Berde CB. Pain Assessment. In: Essentials of Pain Medicine. Elsevier; 2018. 39–46.e1.

[45] Stevens FL, Hurley RA, Taber KH (2011). Anterior cingulate cortex: unique role in cognition and emotion. J Neuropsychiatry Clin Neurosci.

[46] Jin J, Delaparte L, Chen HW, DeLorenzo C, Perlman G, Klein DN, et al. Structural Connectivity Between Rostral Anterior Cingulate Cortex and Amygdala Predicts First Onset of Depressive Disorders in Adolescence. Biol Psychiatry Cogn Neurosci Neuroimaging; 2021. 10.1016/j.bpsc.2021.01.012 33610811

[47] Heuer A, Ohl S, Rolfs M (2020). Memory for action: a functional view of selection in visual working memory. Visual Cognition.

[48] Aagten-Murphy D, Bays PM (2019). Functions of Memory Across Saccadic Eye Movements. Curr Top Behav Neurosci.

[49] van Veen SC, Kang S, van Schie K, On EMDR (2019). Measuring the working memory taxation of various types of eye (non-)movement conditions. J Behav Ther Exp Psychiatry.

[50] Salthouse TA. Reaction Time. In: Encyclopedia of Gerontology. Elsevier; 2007. pp. 407–10.

